# The current clinical landscape of neonatal respiratory failure in Jiangsu Province of China: patient demographics, NICU treatment interventions, and patient outcomes

**DOI:** 10.1186/s12887-024-04741-y

**Published:** 2024-04-25

**Authors:** Na Wang, Ke-Yu Lu, Shan-Yu Jiang, Hong-Wei Wu, Rui Cheng, Zhao-Jun Pan, Huai-Yan Wang, on behalf of the Jiangsu Provincial Collaborative Study Group for Neonatal Respiratory Diseases, Keyu Lu, Huaiyan Wang, Shanyu Jiang, Zhaojun Pan, Hongwei Wu, Zuming Yang, Jie Shao, Shuping Han, Zhengying Li, Yan Xu, Li Ye, Xinping Wu, Hong Li, Guihua Shu, Jinlan Cai, Jinjun Zhou, Xiaoping Yin, Xiaoqing Chen, Songlin Liu, Mengzhu Yu, Yan Gao, Zhidan Bao, Mei Xue, Li Huang, Haiying Li, Lei Song, Wei Wu, Huai Xu, Hongxin Li

**Affiliations:** 1 Department of Neonatology, The Affiliated Suqian First People’s Hospital of Nanjing Medical University, Suqian, Jiangsu China; 2 Department of Neonatology, Children’s Hospital of Nanjing Medical University, Gulou District, No.72, Guangzhou Road, Nanjing, 210008 Jiangsu China; 3 Department of Neonatology, Wuxi Maternity and Child Health Care Hospital, Wuxi, Jiangsu, China; 4 Department of Neonatology, Xuzhou Children’s Hospital Affiliated to Xuzhou medical University, Xuzhou, Jiangsu China; 5 Department of Neonatology, Huai’an maternal and child health care center, Huai’an City, No. 104, Renmin South Road, Qingpu District, Jiangsu, 223001 China; 6 Department of Neonatology, Changzhou Maternity and Child Health Care Hospital, Nanjing Medical University, No.16 Dingxiang Road, Changzhou, 213003 Jiangsu China

**Keywords:** Neonatology, Respiratory failure, Morbidity, Mortality, Neonatal intensive care

## Abstract

**Introduction:**

Neonatal respiratory failure (NRF) is a serious condition that often has high mortality and morbidity, effective interventions can be delivered in the future by identifying the risk factors associated with morbidity and mortality. However, recent advances in respiratory support have improved neonatal intensive care units (NICUs) care in China. We aimed to provide an updated review of the clinical profile and outcomes of NRF in the Jiangsu province.

**Methods:**

Infants treated for NRF in the NICUs of 28 hospitals between March 2019 and March 2022 were retrospectively reviewed. Data collected included baseline perinatal and neonatal parameters, NICU admission- and treatment-related data, and patient outcomes in terms of mortality, major morbidity, and survival without major morbidities.

**Results:**

A total of 5548 infants with NRF were included in the study. The most common primary respiratory disorder was respiratory distress syndrome (78.5%). NRF was managed with non-invasive and invasive respiratory support in 59.8% and 14.5% of patients, respectively. The application rate of surfactant therapy was 38.5%, while that of neonatal extracorporeal membrane oxygenation therapy was 0.2%. Mortality and major morbidity rates of 8.5% and 23.2% were observed, respectively. Congenital anomalies, hypoxic-ischemic encephalopathy, invasive respiratory support only and inhaled nitric oxide therapy were found to be significantly associated with the risk of death. Among surviving infants born at < 32 weeks of gestation or with a birth weight < 1500 g, caffeine therapy and repeat mechanical ventilation were demonstrated to significantly associate with increased major morbidity risk.

**Conclusion:**

Our study demonstrates the current clinical landscape of infants with NRF treated in the NICU, and, by proxy, highlights the ongoing advancements in the field of perinatal and neonatal intensive care in China.

## Introduction

Neonatal respiratory failure (NRF) is a serious clinical condition, and is often a sequelae of respiratory conditions such as respiratory distress syndrome (RDS), meconium aspiration syndrome (MAS), transient tachypnoea of the newborn (TTN), infections, and asphyxia. The prevalence of NRF is notable in the neonatal intensive care unit (NICU) setting, and has been considered both a clinical and economic burden [1]. Based on the earliest epidemiological study conducted by the Chinese Collaborative Study Group for Neonatal Respiratory Diseases between 2004 – 2005, NRF was reported with an incidence of 13.2% across 23 major tertiary hospitals, and an overall mortality rate of up to 32.1% despite 67% of the NICUs being located in the more economically affluent southeastern regions of China [2]. A multi-center study in 2007 – 2008 involving 14 tertiary NICUs in Hebei, a province of moderate economic development, observed an incidence and mortality rate of 16.7% and 31.4%, respectively [3]. A similar study in the United States from the same period [4] involved 1011 near-term newborns (≥ 34 gestational weeks) requiring NICU respiratory support, and found RDS (43.2%) as the most common lung disease. However, compared to the aforementioned studies from China, a considerably lower mortality rate of 5% was observed.

Nonetheless, recent advancements in respiratory and ventilatory support, including non-invasive ventilation techniques [5], surfactant therapy for MAS [6], inhaled nitric oxide (iNO) therapy for pulmonary hypertension [7], and neonatal extracorporeal membrane oxygenation (ECMO) [8], have resulted in improvements in NICU care in China. Such developments in the field, and their effects on NRF outcome, have not been considered in the literature [9–11]. Moreover, current studies on the NICU outcomes of NRF are largely focused on mortality [12, 13]. Considering the improvements in survival prognosis of NRF in recent years, it may be reasonable to expect an increase in disability rate among survivors [14–17]. The morbidity profile and the prospect of survival without major morbidities of such infants thereby remains to be elucidated.

As such, our study aimed to provide an updated review on the clinical landscape of NRF in China, with particular attention paid to survival and morbidity outcomes. Patient characteristics and NICU treatment interventions were further explored to investigate the risk factors for mortality and major morbidity.

## Materials and methods

### Patient selection and study design

Newborns with NRF treated in 28 NICUs in the Jiangsu province of China between March 2019 and March 2022 were retrospectively reviewed. Among the hospitals included, 9 were maternal and child health centers, 5 were children hospitals, and 14 were tertiary general hospitals. NRF was defined as a respiratory disorder with hypoxemia within the first 7 days of life requiring any form of NICU-based respiratory support for at least 24 hours [2]. The inclusion criterion: infants who require admission to the NICU at birth and meet the definition of NRF. The exclusion criteria included missing data on respiratory support methods and outcomes and severe congenital anomalies resulting in death within 48 hours.

This study was approved by the Medical Ethics Committee of the Children's Hospital of Nanjing Medical University (ethics number: 202004037-1).

### Data collection

Data were collected from the clinical records of patients. Among infants who required interhospital transfer, all clinical records were considered unless in cases of transfer within the selected 28 hospitals of the study, wherein records from the initial hospital were omitted to avoid duplicates in data.

Baseline patient characteristics included perinatal data such as maternal age, antenatal steroid use, pregnancy-related complications, premature rupture of membranes of > 24 hours, delivery method, and delivery room resuscitation, as well as neonatal parameters such as gestational age, birth weight, congenital anomalies, and Apgar score. NICU-related data included the primary respiratory disease and types of treatment interventions. Patient outcomes were evaluated in terms of mortality rate, major morbidity rate, and survival without major morbidities rate. The end of morbidity and mortality follow-up was at hospital discharge.

All patients were classified according to birth weight (BW) for subsequent analyses. The patient groups involved infants with extremely low BW, <1000 g; very low BW, 1000 – 1499 g; low BW, 1500 – 2499 g; normal BW, 2500 – 3999 g; and high BW, ≥ 4000 g. For the description of the mortality and morbidity of NRF patients by maturity status, infants with NRF were stratified into: < 32 weeks or weighted weight < 1500 g at birth; ≥ 32 weeks and weighed ≥ 1500g at birth.

### Definitions and diagnostic criteria

RDS was diagnosed based on progressive dyspnea after birth and severe hypoxic respiratory failure; in secondary RDS patients severe respiratory failure with severe hypoxia or infection. Both primary and secondary RDS cases were supported by imaging evidence obtained through chest radiograph or pulmonary ultrasound [18–20]. Pneumonia/sepsis was considered in the presence of clinical or culture evidence of infection [13, 21]. TTN was defined as shortness of breath caused by inadequate lung fluid clearance, with supportive chest X-ray findings of interstitial fluid accumulation and atelectasis [22]. Apnea was defined as cessation of breathing resulting in pathological changes in heart rate ( < 100 beats per minute) and oxygen saturation (respiratory arrest for 20 seconds or longer, often accompanied by cyanosis) [23]. MAS was defined as respiratory distress in a neonate born through meconium-stained amniotic fluid with symptoms which cannot be otherwise explained [24]. Persistent pulmonary hypertension of the newborn (PPHN) was defined as severe hypoxemia accompanied by corresponding chest X-ray and cardiac ultrasound findings [25]. Hypoxic-ischemic encephalopathy (HIE) was defined as hypoxic-ischemic brain damage caused by perinatal asphyxia [26].

Major morbidities referred to any of the following diseases: moderate-to-severe bronchopulmonary dysplasia (BPD, defined as the necessity for oxygen and/or positive pressure at 36 weeks postmenstrual age or discharge) [27], ≥ stage II necrotizing enterocolitis (NEC, modified Bell stage IIA or greater) [28], ≥ grade III intraventricular hemorrhage or periventricular leukomalacia (IVH/PVL, based on the most severe head ultrasonography findings prior to hospital discharge, transfer, or death) [29], ≥ stage III retinopathy of prematurity (ROP) or ROP requiring treatment [30], and late-onset sepsis (LOS, occurring after 72 hours of birth, defined as positive blood culture or the need for antibiotic treatment for ≥ 5 days) [14]. Coagulase-negative Staphylococcus identified from hemoculture was generally regarded as contaminated unless clinical symptoms and other infection indicators suggest the presence of sepsis caused by this Staphylococcus. Furthermore, the screening personnel responsible for IVH and ROP were highly qualified ultrasound physicians and ophthalmologists.

### Statistical analysis

All statistical analyses were performed using the IBM SPSS Statistics 26 software. Continuous variables are expressed as either mean ± standard deviation (SD) or median and quartile range (25th to 75th percentile). The Mann-Whitney U test was used for comparison between 2 groups, while the Kruskal-Wallis H test was used for comparison between multiple groups. Categorical variables are expressed as number and percentage (%). The Chi-square or Fisher’s exact test was used for comparison between groups. Factors potentially associated with death or major morbidities were identified using univariable logistic regression analysis. Risk factors with a probability (*P)* value less than 0.10 by univariate analysis were included in the subsequent multivariate analysis. The crude and adjusted odds ratio (OR) and 95%CI of the identified variables were estimated. *P* < 0.05 was considered statistically significant.

## Results

### Study population and baseline data

A total of 5723 cases of NRF were reported during the study period, accounting for 9.8% of the total number of NICU admissions (*n* = 58,398). Among them, 139 were excluded, of which 110 had missing data, 16 were duplicate cases due to transfers within the collaborative group, and 13 died within 48 hours. A total of 5584 cases were eventually included in the study. All baseline perinatal and neonatal data are presented in Table 1.

**Table 1:** Baseline perinatal and neonatal characteristics of infants with NRF according to birth weight

	**Total** **(** ***n*** ** = 5584)**	**BW, g**					
		**< 1000** **(** ***n*** ** = 314)**	**1000 – 1499** **(** ***n*** ** = 1186)**	**1500 – 2499** **(** ***n*** ** = 2433)**	**2500 – 3999** **(** ***n*** ** = 1535)**	**≥ 4000** **(** ***n*** ** = 116)**	***P*** **-value**
Gestational age (week)	33.4 ± 3.6	27.8 ± 1.9^*^	30 ± 1.8^*^	32.9 ± 1.8^*^	37.5 ± 2.2	39.2 ± 1.6^*^	< 0.001
BW (g)	2105 ± 840	859 ± 100^*^	1274 ± 140^*^	1941 ± 285^*^	3088 ± 403	4365 ± 477^*^	< 0.001
Male, n (%)	3347(59.9)	175(56)^*^	650(54.8)^*^	1438(59.1)^*^	999(64.9)	85(73.3)	< 0.001
Cesarean delivery, n(%)	3664(65.60)	158(50.60)^*^	711(59.90)^*^	1688(69.30)	1081(66.20)	89(76.70)	< 0.001
Fetal distress, n(%)	564(11.2)	34(12.6)	112(11.0)^*^	206(9.2)^*^	193(13.8)	19(3.8)	< 0.001
Congenital anomalies, n(%)	103(1.8%)	11(3.5%)	18(1.5%)	39(1.6%)	32(2%)	3(2.5%)	0.127
Antenatal steroid use^a^, n(%)	1594(33.2)	126(46.8)^*^	519(51.0)^*^	830(39.5)^*^	117(8.9)	2(2.0)^*^	< 0.001
Gestational diabetes, n(%)	907(16.2)	39(12.5)	205(17.2)	381(15.6)	240(15.6)	42(36.2)^*^	< 0.001
Gestational hypertension, n(%)	770(13.7)	89(28.5)^*^	226(19)^*^	328(13.4)^*^	117(7.6)	10(8.6)	< 0.001
Premature rupture of membrane > 24 h, n(%)	550(9.8)	31(9.9)^*^	159(13.4)^*^	288(11.8)^*^	67(4.3)	5(4.3)	< 0.001
Apgar score at 1 min	8(7, 9)	6(4, 8)^*^	8(6, 8)^*^	8(7, 9)^*^	9(8, 9)	8(7, 9)^*^	< 0.001
Apgar score at 5 min	9(8, 10)	8(6, 9)^*^	8(8, 9)^*^	9(8, 10)^*^	9(8, 10)	9(8, 10)	< 0.001
PS in delivery room, n(%)	54(0.9)	4(1.2)	19(1.6)	18(0.7)	12(0.7)	1(0.8)	0.127
Positive pressure ventilation in the delivery room, n(%)	1096(19.6)	105(33.6)^*^	295(24.8)^*^	420(17.2)	252(16.3)	24(20.6)	< 0.001
Endotracheal intubation in delivery room, n(%)	475(8.5)	81(25.9)^*^	114(9.6)^*^	129(5.3)^*^	138(8.9)	13(11.2)	< 0.001
Chest compressions in the delivery room, n(%)	235(4.2)	29(9.2)^*^	62(5.2)	68(2.7)^*^	64(4.1)	12(10.3)^*^	< 0.001

*Abbreviations: NRF* Neonatal respiratory failure, *BW* Birth weight, *PS* Pulmonary surfactant

*P*-values derived from Kruskal-Wallis H or Chi-square test

^*^Statistically significant difference (*P* < 0.05) compared to the normal BW group (2500 – 3999 g)

^a^“Antenatal steroid use” refer to complete or partial antenatal steroid use

The mean gestational age was 33.4 ± 3.6 weeks, and 59.9% of the patients were male. The mean BW was 2105 ± 840 g. Extremely low, very low, low, normal, and high BWs were reported in 5.5%, 21.2%, 43.5%, 27.5%, and 2% of the patients, respectively.

Cesarean delivery was performed in 65.6% of cases. Fetal distress was reported in 11.2% of patients, antenatal steroid administration was reported in 33.2%, and congenital anomalies was reported in 1.8% (*n*=103). These cases mainly included congenital heart disease, gastrointestinal developmental abnormalities, renal developmental abnormalities, congenital lung malformations, esophagotracheal fistula, congenital diaphragmatic hernia, Pierre Robin Sequence, chromosomal abnormalities. The rates of gestational diabetes, gestational hypertension, and premature rupture of membranes were 16.2%, 13.7%, and 9.8%, respectively. Compared to the normal BW group, the high BW group demonstrated significantly higher prevalence of gestational diabetes (15.6% vs. 36.2%, *P* < 0.05), while the extremely low, very low, and low BW groups showed significantly higher prevalence of gestational hypertension (7.6% vs. 28.5%, 19.0%, and 13.4%, respectively; *P* < 0.05) and premature rupture of membranes (4.3% vs. 9.9%, 13.4%, and 11.8%, respectively; *P* < 0.05). Among different groups there was no significant difference in the incidence of congenital anomalies.

The median 1- and 5-minute Apgar scores were 8 (7,9) and 9 (8,10), respectively. Compared to the normal BW group, the extremely low, very low, and low BW groups demonstrated significantly lower 1-minute Apgar scores (*P* < 0.05). Delivery room surfactant therapy was performed in 0.9% of cases, with no significant difference observed between the BW groups (*P* = 0.127). Significantly higher rates of delivery room resuscitation was observed in the extremely low BW group compared to the normal BW group, including endotracheal intubation (25.9% vs. 8.9%, *P* < 0.05) and cardiac compression (9.2% vs. 4.1%, *P* < 0.05).

### NICU-related data

All NICU admission- and treatment-related data are presented in Table 2.

**Table 2:** NICU admission- and treatment-related data

	**Total** **(** ***n*** ** = 5584)**	**BW, g**					
		**< 1000** **(** ***n*** ** = 314)**	**1000 – 1499** **(** ***n*** ** = 1186)**	**1500 – 2499** **(** ***n*** ** = 2433)**	**2500 – 3999** **(** ***n*** ** = 1535)**	**> 4000** **(** ***n*** ** = 116)**	***P*** **-value**
**Underlying respiratory disorder**							
RDS, n(%)	2945(52.7)	245(78.5)^*^	824(69.4)^*^	1371(56.4)^*^	482(31.4)	23(19.8)^*^	< 0.001
Pneumonia/sepsis, n(%)	2028(36.3)	91(29.1)^*^	352(29.6)^*^	775(31.8)^*^	755(49.1)	55(47.4)	< 0.001
TTN, n(%)	515(9.2)	1(0.3)^*^	27(2.2)^*^	232(9.5)^*^	232(15)	23(29.8)	< 0.001
HIE, n(%)	158(2.8)	1( 0.3)^*^	7(1186, 0.5)^*^	28(1.1)^*^	107(6.9)	15(12.9)^*^	< 0.001
Apnea, n(%)	145(2.6%)	11(3.5%)^*^	75(6.3%)	47(1.9%)^*^	12(0.8%)	0(0)	< 0.001
MAS, n(%)	95(1.7)	0(0)^*^	0(0)^*^	7(0.2)^*^	81( 5.2)	6(5.1)	< 0.001
PPHN, n(%)	101(1.8)	1(0.3)^*^	6(0.5)^*^	12(0.4)^*^	74( 4.8)	8(6,8)	< 0.001
**Treatment interventions**							
Non-invasive respiratory support only, n(%)	3341(59.8)	87(27.8)^*^	742(62.5)^*^	1801(74)^*^	665(43.2)	46(39.6)	< 0.001
CPAP initial, n(%)	2843(50.9)	66(21)	595(50.2)	1580(64.9)	564(36.7)	38(32.7)	< 0.001
NIPPV initial, n(%)	213(3.8)	14(4.5)	79(6.7)	91(3.7)	26(1.7)	3(2.6)	< 0.001
BiPAP initial, n(%)	217(3.9)	6(1.9)	51(4.3)	100(4.1)	57(3.7)	3(2.6)	0.314
HFNC initial, n(%)	64(1.1)	1(0.3)	17(1.4)	27(1.1)	17(1.1)	2(1.7)	0.536
NHFOV initial, n(%)	4(0.1)	0(0)	0(0)	3(0.1)	1(0.1)	0(0)	0.722
Non-invasive and invasive respiratory support, n(%)	1431(25.6)	154(49.3)^*^	359(30.2)	473(19.4)^*^	415(27.1)	28(24.1)	< 0.001
Invasive respiratory support only, n(%)	812(14.5)	71(22.7)^*^	85(7.1)^*^	159(6.5)^*^	455(29.6)	42(36.2)	< 0.001
Conventional mechanical ventilation, n(%)	661(11.8)	56(17.8)	64(5.4)	123( 5.0)	382(24.9)	36(31)	0.724
High frequency mechanical ventilation, n (%)	180(3.2)	18(5.7)	14(1.2)	27(1.1)	108(7.0)	13(11.2)	0.684
A repeat mechanical ventilation, n (%)	195(8.7)	77(34.4)^*^	73(16.6)^*^	23(4.8)	19(2.2)	2(2.9)	< 0.001
Escalation to mechanical ventilation from non-invasive ventilation, n(%)	544(11.4)	90(37.3)^*^	199(18.1)^*^	163(7.2)	84(7.8)	8(10.8)	< 0.001
PS (overall), n(%)	2151(38.5)	226(72.4)^*^	647(54.5)^*^	849(34.8)^*^	404(26.2)	25(21.5)	< 0.001
PS for RDS, n(%)	1824(61.9)	193(78.8)^*^	539(65.4)	759(55.4)^*^	317(65.8)	16(69.6)	< 0.001
PS for MAS, n(%)	28(32.6)	2(33.3)	6(33.3)	14(31.1)	6(40)	0(0)	0.845
iNO therapy, n(%)	153(2.7)	11(3.5)	18(1.5)^*^	18(0.7)^*^	95(6.1)	11(9.4)	< 0.001
Caffeine therapy, n(%)	1954(35.3)	254(81.1)^*^	864(73.4)^*^	762(31.5)^*^	71(4.7)	3(2.6)	< 0.001
ECMO, n(%)	12(0.2)	1(0.3)	4(0.3)	5(0.2)	1(0.06)	1(0.8)	0.301

*Abbreviations: NICU* Neonatal intensive care unit, *RDS* Respiratory distress syndrome, *TTN* Transient tachypnoea of the newborn, *HIE* Hypoxic-ischemic encephalopathy, *MAS* Meconium aspiration syndrome, *PPHN* Persistent pulmonary hypertension of the newborn, *CPAP* Continuous positive airway pressure, *NIPPV* Non-invasive positive pressure ventilation, *BiPAP* Bi-level positive airway pressure, *HFNC* High-flow nasal cannula, *NHFOV* Non-invasive high-frequency oscillatory ventilation, *PS* Pulmonary surfactant, *iNO* Inhaled nitric oxide, *ECMO* Extracorporeal membrane oxygenation, *BW* Birth weight

*P*-values derived from Chi-square test

^*^Statistically significant difference (*P* < 0.05) compared to the normal BW group (2500 – 3999 g)

RDS was reported in 52.7% of cases, with significant decrease observed with BW (*P* < 0.05). The remaining underlying respiratory conditions included pneumonia/sepsis (36.3%), TTN (9.2%), HIE (2.8%), apnea (2.6%), MAS (1.7%), and PPHN (1.8%).

In the NICU setting, 59.8% (*n* = 3341) received non-invasive respiratory support only, 14.5% (*n* = 812) received invasive respiratory support only, and 25.6% (*n* = 1431) received both. The most common non-invasive technique employed was continuous positive airway pressure (CPAP initial, 50.9%), followed by non-invasive positive pressure ventilation (NIPPV initial, 3.8%), bilevel positive airway pressure (BiPAP initial, 3.9%), high-flow nasal cannula (HFNC initial, 1.1%), and non-invasive high-frequency oscillatory ventilation (NHFOV initial, 0.1%). Compared to the normal BW group, the application rate of non-invasive respiratory support only, in general, was significantly lower in the extremely low BW group (43.2% vs. 27.8%,* P* < 0.05), but was significantly higher in the very low and low BW groups (43.2% vs. 62.5% and 74.0%, respectively; both *P* < 0.05). Conventional and high-frequency invasive mechanical ventilation was employed in 11.8% and 3.2% of patients, respectively. Repeat mechanical ventilation was required in 8.7% of patients. Escalation to mechanical ventilation from non-invasive ventilation was reported in 11.4% of patients. Similarly, significantly higher rates were observed in the extremely low BW group compared to the normal BW group (37.3% vs 7.8%, *P* < 0.05).

Surfactant therapy was performed in 38.5% of patients, of whom 61.9% and 32.6% had RDS and MAS, respectively. iNO therapy was employed in 2.7% of patients, while caffeine therapy was utilized in 35.3%. ECMO was initiated in 0.2% of patients.

### Morbidity and mortality

The outcome indicators at discharge of all patients are presented in Table 3.

**Table 3:** Morbidity and mortality of infants with NRF

	**Total** **(** ***n*** ** = 5584)**	**BW, g**					
		**< 1000** **(** ***n*** ** = 314)**	**1000 – 1499** **(** ***n*** ** = 1186)**	**1500 – 2499** **(** ***n*** ** = 2433)**	**2500 – 3999** **(** ***n*** ** = 1535)**	**> 4000** **(** ***n*** ** = 116)**	***P-*** **value**
Mortality, n(%)	475(8.5)	100(32)^*^	130(10.9)	99(4)^*^	130(8.4)	16(13.7)	< 0.001
Survival without major morbidities, n(%)	3810(68.2)	84(26.8)^*^	692(58.3)^*^	1860(76.4)^*^	1093(71.2)	81(69.8)	< 0.001
Major morbidities, n(%)	1299(23.2)	128(40.8)^*^	364(30.7)^*^	474(19.5)	314(30.5)	19(16.4)	< 0.001
BPD ≥ moderate, n(%)	210(3.8)	74(23.6)^*^	107(9.0)^*^	24(1.0)	4(0.3)	0(0)	< 0.001
NEC ≥ stage 2, n(%)	94(1.7)	21(6.7)^*^	47(4.0)^*^	21(0.9)	4(0.3)	1(0.9)	< 0.001
IVH/PVL ≥ grade 3, n(%)	56(1)	21(6.7)^*^	24(2.0)^*^	8(0.3)	3(0.2)	0(0)	< 0.001
ROP ≥ stage 3, n(%)	74(1.3)	17(5.4)^*^	42(3.5)^*^	14(0.6)	1(0.1)	0(0)	< 0.001
LOS, n(%)	1214(21.7)	101(32.2)^*^	279(23.5)	460(18.9)^*^	348(22.7)	26(22.4)	< 0.001

*Abbreviations: NRF* Neonatal respiratory failure, *BPD* Bronchopulmonary dysplasia, *NEC* Necrotizing enterocolitis, *IVH/PVL* Intraventricular hemorrhage/periventricular leucomalacia, *ROP* Retinopathy of prematurity, *LOS* Late-onset sepsis, *BW* Birth weight

*P*-values derived from Chi-square test

^*^Statistically significant difference (*P* < 0.05) compared to the normal BW group (2500 – 3999 g)

**Fig. 1:**
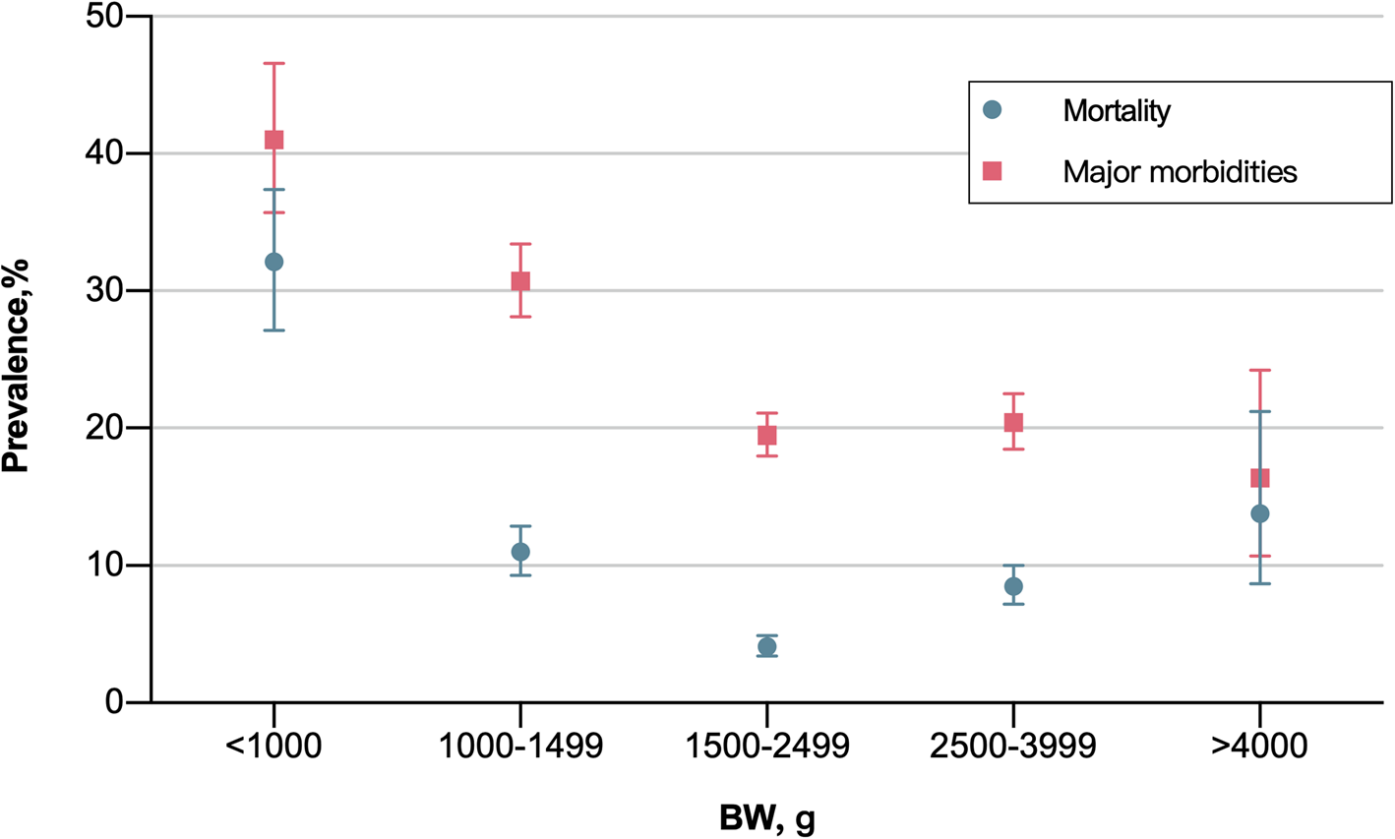
Mortality and major morbidities in NRF infants according to birth weight. *Abbreviation: NRF* neonatal respiratory failure, *BW* birth weigh

A mortality rate of 8.5% was observed. Compared to the normal BW group, significantly higher and lower mortality rates were demonstrated in the extremely low (8.4% vs. 32%, *P* < 0.05) and low BW groups (8.4% vs. 4%, *P* < 0.05), respectively (Fig. 1). Major morbidities were observed in 23.2% of patients. The extremely low BW group demonstrated significantly higher overall rate of major morbidity compared to the normal BW group (Fig. 1). Compared to the normal BW group, significantly lower survival without major morbidities rates were observed in the extremely low and very low BW groups (71.2% vs. 26.3% and 58.3%, respectively; *P* < 0.05), while significantly higher rates were seen in the low BW group (71.2% vs. 76.4%, *P* < 0.05). The minimum BWs for overall and survival without major morbidities were observed to be 570 g and 630 g, respectively.

### Risk factors for mortality and morbidity

Through univariate logistic regressions, we identified factors associated with death and major morbidities during the prenatal, intrapartum, and postpartum stages. Subsequently, we conducted further multivariable regression model analysis of these factors. All multivariate logistic regression analysis results were shown in Tables 4 and 5.

**Table 4:** Risk factors for mortality in infants with NRF

	**Unadjusted OR(95% CI)**	**Adjusted OR(95% CI)**
Higher gestational age	0.92(0.89, 0.94)	0.89(0.82, 0.96)
Higher BW	1.00(1.00, 1.00)	1.00(0.99, 1.00)
Congenital anomalies	5.63(3.69, 8.58)	8.54(5.03, 14.49)
Cesarean delivery	0.62(0.51, 0.75)	0.73(0.57, 0.94)
Higher Apgar score at 1 min	0.75(0.72, 0.78)	0.85(0.78, 0.94)
HIE	2.85(1.91, 4.25)	2.48(1.35, 4.56)
Non-invasive respiratory support only	0.15(0.12, 0.19)	0.40(0.29, 0.57)
Invasive respiratory support only	9.18(7.51, 11.21)	6.85(5.01, 9.36)
PS	1.61(1.33, 1.95)	0.80(0.61, 1.06)
iNO therapy	6.49(4.59, 9.17)	3.81(2.24, 6.49)
Caffeine therapy	1.09(0.93, 1.33)	0.68(0.50, 0.94)
**Subgroup analysis(< 32 weeks or < 1500 g at birth)**		
Higher gestational age	0.63(0.59, 0.68)	0.82(0.75, 0.93)
Higher BW	0.99(0.99, 0.99)	0.99(0.99, 0.99)
Congenital anomalies	4.85(2.40, 9.82)	7.84(3.38, 18.17)
Higher Apgar score at 1 min	0.74(0.70, 0.78)	0.86(0.77, 0.96)
Invasive respiratory support only	16.33(11.88, 22.44)	8.83(5.83, 13.87)
PS	2.18(1.66, 2.86)	0.82(0.56, 1.19)
iNO therapy	9.57(4.85, 18.85)	5.84(2.51, 13.62)
Caffeine therapy	0.63(0.48, 0.83)	0.44(0.31, 0.63)
**Subgroup analysis( ≥ 32 weeks and ≥ 1500 g at birth)**		
Congenital anomalies	7.08(4.15, 12.08)	8.66(4.00, 18.75)
Non-invasive respiratory support only	0.12(0.87, 1.81)	0.41(0.22, 0.74)
Invasive respiratory support only	9.82(7.29, 13.22)	4.25(2.56, 7.06)
iNO therapy	7.29(4.77, 11.13)	2.19(1.05, 4.54)

*Abbreviations: NRF* Neonatal respiratory failure, *BW* Birth weight, *HIE* Hypoxic-ischemic encephalopathy, *PS* Pulmonary surfactant, *iNO* Inhaled nitric oxide, *CI* Confidence interval, *OR* Odds ratio

**Table 5:** Risk factors for major morbidity among surviving infants born at < 32 weeks of gestation or with a birth weight < 1500 g

	**Unadjusted OR(95% CI)**	**Adjusted OR(95% CI)**
Higher gestational age	0.70(0.66, 0.74)	0.85(0.70, 1.02)
Higher BW	0.99(0.99, 0.99)	0.99(0.99, 1.00)
Higher Apgar score at 1 min	0.90(0.86, 0.94)	1.16(0.97, 1.39)
Higher Apgar score at 5 min	0.85(0.80, 0.91)	0.86(0.70, 1.07)
A repeat mechanical ventilation	2.30(1.89, 2.81)	2.26(1.81, 2.82)
PS	1.81(1.50, 2.19)	0.98(0.56, 1.73)
Caffeine therapy	2.93(2.31, 3.71)	2.49(1.35, 4.59)

*Abbreviations: NRF* Neonatal respiratory failure, *BW* Birth weight, *PS* Pulmonary surfactant, *CI* Confidence interval, *OR* Odds ratio

Congenital anomalies, HIE, invasive respiratory support only and iNO therapy were found to be significantly associated with the risk of death. Higher gestational age, birth weight and 1-minute Apgar score, as well as cesarean delivery, non-invasive respiratory support only and caffeine therapy, were associated with a reduced risk of death.

Subgroup analysis showed that among infants with < 32 weeks or < 1500 g at birth, congenital anomalies, invasive respiratory support only and iNO therapy were found to be significantly associated with the risk of death. Higher gestational age, birth weight, 1-minute Apgar score and caffeine therapy were associated with a reduced risk of death. Among infants with ≥ 32 weeks and ≥ 1500g at birth, Congenital anomalies, invasive respiratory support only and iNO therapy were found to be significantly associated with the risk of death. Non-invasive respiratory support only was associated with a reduced risk of death.

Given the significantly higher morbidity rates observed among surviving infants born at < 32 weeks of gestation or with a birth weight < 1500 g, a subgroup analysis was performed, as shown in Table 5. Caffeine therapy and a repeat mechanical ventilation were demonstrated to significantly associate with increased major morbidity risk.

## Discussion

Our multicenter study highlights the current clinical landscape of NRF in the economically developed areas of eastern China. Consistent with the ongoing advancements in respiratory support techniques in the NICU, our findings demonstrate the current status of mortality secondary to NRF in recent years. It also describes survival with or without major morbidities under different BW groups. Overall, our study reflects the impact of a wide-range of factors, from perinatal and neonatal parameters to care measures in the delivery room and NICU setting, on the prognosis of infants with NRF. For moderate and late preterm infants, congenital anomalies and NICU interventions emerged as significant predictors of mortality. However, among very preterm infants, birth conditions such as gestational age, birth weight, and Apgar score, along with NICU interventions, were identified as significant risk factors for both mortality and major morbidity.

Our findings demonstrated that overall mortality of NRF was 8.5%, compared to 32.1% of 15 years ago [2], reflecting the continuous development in NICU management in China. The five major morbidities were BPD ≥ moderate, NEC ≥ stage 2, IVH/PVL ≥ grade 3, ROP ≥ stage 3, and LOS [14, 31]. Importantly, the incidence of LOS was noted to be the highest among the major morbidities (21.7%), with no significant downward trend observed compared to reports of a similar study in 2012 by Wang et al. (23.5%) [13]. Given the known correlation between inappropriate antibiotic use and mortality risk among culture-negative infants [32], further studies are warranted to clarify the indications and approach to antibiotic therapy to improve neonatal outcomes.

In addition, we found that normal BW infants demonstrated higher mortality rates compared to low BW infants. Correspondingly, the morbidity rates of normal BW infants were observed to be higher. The tendency for NRF in normal BW infants to be due to more serious diseases may be a reason for this. For instance, fetal distress at birth, and the resultant need for endotracheal intubation and cardiac compression in the delivery room setting, were observed to be more prevalent in normal BW infants of our study. This is consistent with the study by Arnon et al., which demonstrated an association between the need for delivery room cardiopulmonary resuscitation and the risk of adverse outcomes such as death [33]. Therefore, it is important to pursue normal BW and not resist it because of the high mortality rate of NRF.

Our study also demonstrated an increase in the uptake of non-invasive respiratory support techniques compared to previous studies, and a corresponding decrease in the practice of invasive approaches [13]. Among the non-invasive interventions, CPAP was most commonly employed (85%). This is consistent with the 2022 European Consensus Guidelines on the Management of Respiratory Distress Syndrome [34], which advocates for the avoidance of invasive mechanical ventilation in premature infants, and recommends CPAP as first-line intervention for primary and secondary respiratory support [35, 36]. In our study, NIPPV and BiPAP were used in 6.3% and 6.4% of patients, respectively. Advancements in ventilator design have resulted in the ability for NIPPV to deliver pressures comparable to invasive mechanical ventilation. In the systematic review by Ramaswamy et al. comparing different NIV modes for primary respiratory support, synchronized NIPPV was concluded as the most effective intervention in reducing the need for invasive mechanical ventilation or re-ventilation among premature infants [37]. The randomized clinical trial by Zhu et al. found that NHFOV and NIPPV resulted in a significantly lower risk of reintubation compared to CPAP [5]. In contrast, research on BiPAP, particularly its clinical benefits in comparison to CPAP, is currently lacking [34]. Further prospective studies are thereby needed to elucidate the optimal NIV approach for the primary treatment of NRF.

Surfactant therapy was employed in 38.5% of our patients, of whom 61.9% and 32.6% had RDS and MAS, respectively. While the role of surfactant therapy in RDS is well-established [34, 38], its use for the treatment of MAS has only recently gained clinical attention. Our findings corroborated with this, and demonstrated a considerably increased use of surfactant therapy for MAS compared to the previous data (1.8%) [2]. According to the meta-analysis by Hui et al. [39], surfactant lavage significantly reduced the duration for mechanical ventilation without increasing the risk of morbidity. PPHN of the newborn due to MAS has a high morbidity and mortality [40]. Nonetheless, there remains a need to clarify the effects of surfactant therapy on the risk of mortality and PPHN in neonates with MAS.

iNO therapy has demonstrated a role in the management of respiratory failure [41]. In our study, the usage rate of iNO was only 2.7%. However, iNO therapy was limited to infants with evidence of pulmonary hypertension and severe respiratory distress. The reservation of this treatment intervention for neonates in severe respiratory conditions may be related to our findings regarding the correlation between iNO treatment and mortality.

Caffeine therapy was used in 38.5% of our patients, and was found as a significantly protective factor against mortality in NRF. This is consistent with the reports of Lodha et al. [42]. However, a significant correlation between caffeine therapy and morbidity was also observed. This may be due to inherent nature of our patient population, and the tendency of neonates with respiratory failure to be of a more severe clinical state. Caffeine therapy was used in 88.6% of our patients with major morbidities, of whom had an average gestational age and BW of 29 weeks and 1193 g, respectively. Currently, local protocols recommend the administration of caffeine therapy immediately after birth for infants of ≤ 30 gestational weeks or ≤ 1500 g to reduce the risk of adverse outcomes such as BPD. The use of other methylxanthine derivatives instead of caffeine in some NICUs could explain the low percentage of caffeine administration. The high correlation between caffeine use and major morbidities did not necessarily indicate that major morbidities would not occur without the use of caffeine. The underlying reasons for this seemingly paradoxical correlation were linked to the small gestational age, low birth weight, severe condition of newborns with respiratory failure, and the widespread use of caffeine in premature infants with small gestational age. Importantly, evidence has shown the potential of caffeine therapy in conferring greater neurodevelopmental benefits among infants on respiratory support compared to those who are not [43]. Further studies are thus required to evaluate the impact of caffeine in the management of NRF on long-term outcomes such as neurodevelopmental disability.

Compared to adults and children, ECMO support has demonstrated the best prognosis among neonates, with an average survival rate of 75% [44]. Neonatal ECMO has been applied in the context of MAS, PPHN, and RDS [45]. The application rate of ECMO was observed to be low in our study (0.2%), and is considerably lower than that reported in the US (5.0%) [46]. This may reflect the underdevelopment of neonatal ECMO in China, and the lack of best practice guidelines in the field. However, the success rate of ECMO in our study was relatively high (66.7%), with the minimum BW for successful treatment being 990 g. Our study thereby highlights the potential of existing ECMO technology for the management of premature infants with NRF in China.

Our research revealed that in China, in general, newborns admitted to the NICU were promptly provided with active treatment regardless of the type of congenital anomalies or the severity of hypoxic-ischemic encephalopathy (HIE), with the exception of those who succumbed to severe congenital anomalies within 48 hours of birth. Considering the high incidence of congenital anomalies and the adverse neurological outcomes of HIE, quantifying the decision on the extent of treatments or the decision to stop treatments for HIE babies or babies with congenital anomalies requires the consideration of multiple factors. A survey conducted by the domestic respiratory failure group on infants with respiratory failure who stopped treatment showed that the reasons for most parents stopping treatment were not singular, mainly due to serious sequelae and uncontrollable diseases [47]. Combining our research results, when infants with HIE/congenital anomalies failed to show significant clinical improvement despite active treatment, and their parents were concerned about poor long-term prognosis, they may decide to discontinue the treatment. This partly explains why these two factors serve as significant risk factors for mortality.

This study had several limitations. First, our study was retrospective in design, with the possibility of significant patient factors being missed in our analysis. Furthermore, our patients were classified according to BW only, and not gestational age. We believe that this can be justified based on our findings of BW as a significant associating factor for mortality and morbidity on multivariate analysis. However, this resulted in the overlooking of morbidity and mortality trends in different gestational age groups. Further studies are thereby warranted to explore this topic.

## Conclusion

Our study demonstrates the current clinical landscape of infants with NRF treated in the NICU, and, by proxy, highlights that ongoing advancements in the field of perinatal and NICU care in China. Our findings suggest that strengthening perinatal care, in addition to neonatal intensive care measures such as encouraging non-invasive respiratory support methods and caffeine therapies, can assist in mitigating mortality rates among infants with NRF.

## Data Availability

The datasets used and/or analysed during the current study available from the corresponding author on reasonable request.
